# Level of non-adherence and its associated factors among adults on first-line antiretroviral therapy in Amhara Regional State, Ethiopia

**DOI:** 10.1371/journal.pone.0255912

**Published:** 2021-08-09

**Authors:** Setognal Birara Aychiluhm, Abay Woday Tadesse, Kusse Urmale Mare, Mequannent Sharew Melaku, Ibrahim Mohammed Ibrahim, Osman Ahmed, Oumer Abdulkadir Ebrahim, Mohammed Wagris, Yonatan Menber, Ayesheshim Muluneh Kassa

**Affiliations:** 1 Department of Public Health, College of Medicine and Health Sciences, Samara University, Samara, Ethiopia; 2 Department of Nursing, College of Medicine and Health Sciences, Samara University, Samara, Ethiopia; 3 Department of Health Informatics, Institute of Public Health, College of Medicine and Health Sciences, University of Gondar, Gondar, Ethiopia; 4 Department of Midwifery, College of Medicine and Health Sciences, Samara University, Samara, Ethiopia; 5 Department of Public Health, College of Health Sciences, Bahir Dar University, Bahir Dar, Ethiopia; 6 Dream Science, and Technology College, Dessie, Ethiopia; 7 Dessie Health Science College, Dessie, Ethiopia; Johns Hopkins University, UNITED STATES

## Abstract

**Background:**

In Ethiopia, nearly one-third of people living with human immunodeficiency viruses do not adhere to antiretroviral therapy. Moreover, information regarding non-adherence and its associated factors among adults on first-line antiretroviral therapy in Northeast Ethiopia is limited. Therefore, this study aimed to assess the level of non-adherence and its associated factors among adults on first-line antiretroviral therapy in North Shewa Zone, Amhara Regional State, Ethiopia.

**Methods:**

A facility-based cross-sectional study was conducted on 326 participants selected by systematic random sampling technique from the five randomly selected public health facilities. Data were collected using the questionnaire adapted from the studies conducted previously and the collected data were entered into Epi data version 3.1 and exported to Stata version 14 for further analysis. Multivariable logistic regression analysis was done and an adjusted odds ratio with its corresponding 95% confidence interval was used to declare a statistical significance.

**Results:**

The overall prevalence of non-adherence was 17.4% [95% CI: (12.8%, 21.2%)]. Patients with no formal education [AOR (95% CI) = 5.57 (1.97, 15.88)], those who did not use memory aids to take their medications [AOR (95% CI) = 3.01 (1.27, 7.11)], travel more than 10 kilometers to visit the nearby antiretroviral therapy clinics [AOR (95% CI) = 2.42 (1.22, 25.86)], those who used substance [AOR (95% CI) = 3.57 (1.86, 28.69)], and patients whose medication time interfered with their daily routine activities [AOR (95% CI) = 15.46 (4.41, 54.28) had higher odds of having non-adherence to first-line antiretroviral therapy compared to their counter groups.

**Conclusion:**

The level of non-adherence to first-line antiretroviral therapy was 17.4%, higher compared to WHO’s recommendation. Hence, patients counseling focused on avoiding substance use, use memory aids, and adjusting working time with medication schedule are very crucial. Furthermore, the ministry of health and the regional health bureau with other stakeholders should expand antiretroviral therapy service delivery at health facilities that are close to the community to address distance barriers.

## Background

According to the 2019 global estimate, about 36.2 million adults were living with the human immunodeficiency virus (HIV), of these, 20.6 million were found in Sub-Saharan countries [1–3]. In Ethiopia, more than half a million (722,248) people are living with HIV, of these, one-third (30%) were from Amhara Regional State [4, 5].

The adult HIV prevalence in Ethiopia is 0.9% [6], with 2.9% in urban and 0.4% in rural settings [4, 7, 8]. The incidence of a new infection is also at a steady state [7, 9], below three-fourth of People Living With HIV(PLWHIV) ever enrolled on antiretroviral therapy (ART) in the country [3, 8, 10].

Recent data showed that above 95% adherence to the ART regimen is required for HIV infected patients to reach full viral suppression[11–13], but sustaining its adherence level requires accurate and consistent monitoring activities, and this is a major challenge for sub-Saharan African countries like Ethiopia [14]

In Ethiopia, 5% to 35% of PLWHIV did not adhere to the prescribed ART regimens [15–20] which is far away from the national target of HIV prevention programs [4]. Studies conducted in different settings identified socio-demographic, behavioral, disease characteristics, medication, and health systems-related factors as the common factors affecting adherence to ART regimens [15, 21–26].

In Ethiopia, different strategies like a transformation of a fee-based ART program in 2003 to a free ART program in 2005, decentralization of services to lower-level public health facilities and private hospitals, and capacity building for a service provider on counseling aspect have been tried to improve adherence to ART [27, 28]. However, still maintaining adherence to ART regimens has been one of the challenges of ART programs in the country [17, 29, 30].

Studies conducted previously were focused on clinical determinants of non-adherence rather than looking at the clients’ behavioral aspects. Besides, little is known about the level of non-adherence and its associated factors in the study area. Therefore, this study aimed to determine the level of non-adherence and its associated factors among adults on first-line ART in North Shewa Zone, Amhara Regional State, Ethiopia.

## Methods and materials

### Study setting, design, period, and population

A facility-based cross-sectional study was conducted from 15^th^ March to 15^th^ April 2019 in North Shewa Zone, Amhara Regional State, Ethiopia, which is located 120 kilometers away from Addis Ababa, the capital of Ethiopia. In this Zone, there are thirteen health centers and five public hospitals. In all of the health facilities, the patients have a regular visit to the ART clinic on a monthly follow-up basis.

In this study, out of the ART providing public health facilities in the study area, one referral hospital (Debre Birhan Referral Hospital) and four health centers (Chacha, Debresina, Shewa-Robit, and Debre Brihan Health Centers) were randomly selected. All of the five health facilities are located in urban areas and are providing ART services for people coming from both urban and rural dwellers.

All adults on ART who were at least 18 years of age, able to hear, mentally fit, and being on follow-up for at least 3 months before the data collection period were eligible for this study. While those who were seriously ill during the data collection period were excluded.

### Sample size determination and sampling techniques

For the first specific objective (i.e. to determine the level of non-adherence to first-line antiretroviral therapy among Adults in North Shewa Zone, Amhara Regional State, Ethiopia), the sample size was estimated using a single population proportion formula by considering a 95% confidence level, 5% margin of error, and non-adherence level of 25.8%, taken from the study conducted in Southwest Ethiopia [31].


$$n=\left[\frac{{\left(z\frac{\alpha }{2}\right)}^{2}p(1-p)}{d^{2}}\right]$$

Where, n = required sample size, Zα/2 = critical value for normal distribution at 95% confidence level (1.96), p = expected level of non-adherence, and d = margin of error). Based on the above assumptions, the final sample size for the study was 296.

For the second specific objective (i.e. to identify the factors associated with non-adherence to first-line antiretroviral therapy among adults in North Shewa Zone, Amhara Regional State, Ethiopia), the sample size (118) was estimated using Epi-info version 7.2.1 by considering different variables that have a statistically significant association with non-adherence [20], the ratio of exposed to unexposed to be 1:1, 95% confidence level, and power of 80%. Finally, from the sample sizes estimated for both specific objectives, the minimum largest sample size was 296 (i.e. the sample size of the first objective). Then, with a 10% non-response rate, the final sample size was 326 adults on first-line ART.

Finally, the sample size was proportionally allocated to each health facility based on their preceding one-year adult ART case flow. Then, the sampling fraction was determined by dividing the total number of the eligible population getting services in each facility by the allocated sample size (N/n), which is approximately equal to six for all facilities. Accordingly, every 6^th^ participant was selected using a systematic random sampling technique from the patients’ registration book.

### Study variables

#### Dependent variable

Adherence status to first-line ART that was dichotomized into “non-adhered” and “adhered” was measured by using the patient’s self-reported percentage of missed doses during the last month. Thus, participants who missed more than 5% of the prescribed ART doses based on self-report were considered to have non-adhered to the treatment and otherwise considered to have adhered to the treatment [32, 33]

#### Independent variables

Sociodemographic characteristics (educational level, monthly income, marital status, residence), use of memory aids to take ART drugs, get social support, type of substance used, medication time interfere with daily activities, and distance from ART clinics.

### Definition of terms

#### Social support

A participant was considered as getting social support if he/she gets economic, psychological, and social support from his relative’s/family’s members.

#### Emotional life

The emotional life of the participants was assessed using five (yes/no) questions. Participants who responded correctly to three or more questions were categorized as having a stable emotional life and those who provided a correct response for less than three questions were categorized as having unstable emotional life.

#### Substance use

Participants who reported using at least one substance (alcohol, cigarette, and khat) 7 days before the data collection period were considered as “using a substance” and otherwise considered as “not using a substance”.

### Data collection tools and procedure

The questionnaire was adapted from studies conducted in developing countries [6, 34–37]. The questionnaire consists of sociodemographic, social support, behavioral factors, level of adherence, use of memory aids to take the ART drugs, and history of other medical illnesses. It was initially developed in the English language and translated to the local language (Amharic) and translated back to English to confirm its consistency. The tool was pretested on 5% (16 participants) of sample size other than selected health facilities and some amendments were made based on the pretest findings. Data were collected by trained health professionals who were not working in the ART clinic in the selected health facilities.

### Data management and statistical analysis

The data were cleaned, coded, and entered into Epi data version 3.1 and exported to Stata version 14 for analysis. To assess the association between the dependent variable with each independent variable, bivariable logistic regression analysis was done and those independent variables with a p-value less than 0.25 were considered in the final multivariable logistic regression model. The eligible covariates were socio-demographic characteristics, use of memory aids, distance to ART clinics, social supports, substance use (alcohol, chat chewing, or cigarette smoking), and time of medication taking. The correlation between independent variables was checked using variance inflation factor (VIF) and its value for all variables was less than 5. The model fitness was assessed using the Hosmer-Lemeshow model fitness test (p-value of 0.32). Adjusted odds ratio with its corresponding 95% confidence interval was used to declare a statistical significance.

### Ethical consideration and consent to participate

Ethical clearance was obtained from Research Ethics Review Committee, Dream Science and Technology College (DSTC) with an ethical letter of DSTC/0123/2019. Confidentiality of the information was secured by excluding respondents’ identifiers, such as names, from the data collection format. Finally, written and verbal informed consent was obtained from those who were willing to participate in the study.

## Results

### Participants’ socio-demographic characteristics

In this study, 310 participants were included with a response rate of 95.1%. The mean age of the respondents was 40.3±1.32 years. The median duration on ART was 39 [interquartile range (24–72)] months. Of the total respondents, 193 (62.3%) were residing in rural areas, 60 (19.4%) did not attend formal education. Regarding occupation and religious status, 137 (44.2%) were merchants, and nearly half (48.1%) were orthodox religious followers (**Table 1).**

**Table 1:** Socio-demographic characteristics of participants on first-line ART at public health facilities of North Shewa Zone, Amhara Regional State, Ethiopia, 2019.

Variable	Category	Frequency	Percent
**Educational level**	No formal education	60	19.4
	Elementary	79	25.5
	Secondary	117	37.7
	Tertiary	54	17.4
**Occupational status**	Farmers	93	30.0
	Government employees	31	10.0
	Merchants	137	44.2
	Others*	49	15.8
**Monthly income (in ETB)**	<1500	100	32.2
	≥1500	210	67.8
**Religion**	Orthodox	149	48.1
	Protestant	121	39.0
	Others**	40	12.9
**Marital status**	Single	70	22.6
	Married	90	29.0
	Divorced	14	4.5
	Widowed	136	43.9
**Residence**	Urban	117	37.7
	Rural	193	62.3

Others*(daily labor^20^, Non-governmental organization(NGO) employee^14^, student^15^), Others** (catholic^10^, Muslim^17^, Jehovah witness^6,^ and Waka Feta^7^)

### HIV/AIDS and ART service-related information

Two hundred twenty- six participants (72.9%) heard about HIV/AIDS before their first contact with health care providers, and 199 (64.2%) participants got information about ART from health care providers. Nearly two-thirds (69.7%) of the participants were aware of the benefits of ART before they started treatment and 226 (72.9%) reported as they were benefited from their ART (**Table 2**).

**Table 2:** HIV/AIDS and ART service-related information of adults on first-line ART at public health facilities of North Shewa Zone, Amhara Regional State, Ethiopia, 2019.

Variable	Category	Frequency	Percent
**Heard about HIV infection before illness**	Yes	226	72.9
	No	84	27.1
**Heard about the availability of ART**	Before diagnosis	99	31.9
	During diagnosis	149	48.1
	After diagnosis	62	20.0
**Source of information for ART**	Health care providers	199	64.2
	Radio	38	12.3
	Television	61	19.7
	Others*	12	3.8
**Aware of the benefit of ART when initiated**	Yes	216	69.7
	No	94	30.3
**Place where ART initiated**	Health center	140	45.2
	Hospital	151	48.7
	Others**	19	6.1
**Aware of adherence before starting ART**	Yes	234	75.5
	No	76	24.5
**Adherence in patients understanding**	Taking all prescribed drugs	70	22.6
	Not missing more than 2 doses	56	18.1
	Not missing more than 3 doses	65	21.0
	Not missing more than 4 doses	43	13.9
**Know the current CD4 count**	Yes	264	85.2
	No	46	14.8
**Benefited from ART**	Yes	226	72.9
	No	84	27.1

Others* (peers^5^, religious leaders^3^, and family members^4^), Others** (NGO clinics^11^, and private clinics^8^)

### Behavioral, psycho-social, health service, and ART drug-related information

The main reasons for missed ART doses were living far away from the health facilities (11.9%), forgetting (7.4%), drug side effects (6.8%), and being busy (6.1%). Moreover, 61.0% of the participants reported as they had ART-related side effects predominantly vomiting (24.8%) and diarrhea (18.7%). The study revealed that 60.3% of respondents had a history of substance use (**Table 3**).

**Table 3:** Behavioral, psycho-social, health service, and ART drug-related information among adults on ART at public health facilities of North Shew Zone, Amhara Regional State, Ethiopia, 2019.

Variable	Category	Frequency	Percent
Experienced ART related side effects	Yes	189	61.0
	No	121	39.0
Type of side effects	Skin rash	52	16.8
	Vomiting	77	24.8
	Diarrhea	58	18.7
	Others*	2	0.7
Decision made by patients during side-effects	Discontinue ART till next Appointment	72	23.2
	Continue ART till next Appointment	42	13.5
	Immediately call for health care providers	43	13.9
	Others**	32	10.3
Pills taken by patients per day	1–3 pills	247	79.7
	Above 3 pills	63	20.3
Use memory aids to take ARV drugs	Yes	169	54.5
	No	141	45.5
Type of memory aids commonly used	Pill box	42	13.5
	Written schedule	38	12.3
	Watch alarm	75	24.2
	Family members	11	3.5
	Others***	3	1.0
Feels Comfortable when taking ARV drugs in front of others	Yes	78	25.2
	No	232	74.8
Reasons for not feeling comfortable	Fear of stigma	138	44.5
	Fear of insult from partner	66	21.3
	Others****	28	9.0
Get social support	Yes	121	39.0
	No	189	61.0
Pattern of emotional life	Unstable	110	35.5
	Stable	200	64.5
Medication schedule interfere with daily activity	Yes	168	54.2
	No	142	45.8
Use any substance in the last seven days	Yes	182	58.7
	No	128	41.3
Type of substance used in the last seven days	Alcohol	102	56.0
	Cigarette	39	21.4
	Khat	41	22.5
Missed prescribed doses in the last month	Yes	100	32.3
	No	210	67.7
Number of doses missed in the last one month	0 doses	210	67.7
	1–2 doses	35	11.3
	3 doses	11	3.6
	4 and more doses	54	17.4
Reasons for a missed dose	Being busy	19	6.1
	Due to ART side effects	21	6.8
	Forgetting	23	7.4
	Living far away	37	11.9
Distance from ART clinic	< 10killometers	119	38.4
	10killometers and above	191	61.6

Others* (sever headach^1^, and nausea^1^), Others** (take pain killers^12^, visit private clinic^9^, visit health facility^11^), Others*** (calendar^2^, and partner^8^), Others**** (lack of confidence^16^, and fear of insult from parents^12^)

### Non-adherence level

Of the total respondents, 54 (17.4%) [95% CI: 12.8% to 21.2%] were non-adherent to their prescribed ART drugs.

### Factors associated with non-adherence level

After Adjusting for selected covariates, patients with no formal education had more than five times higher odds of non-adherence compared to those who attended at least secondary education [AOR (95% CI) = 5.57 (1.97, 15.88)]. In this study, patients who did not use memory aids to take their medications were three times more likely to be non-adhered compared to their counterparts [AOR (: 95% CI) = 3.01 (1.27, 7.11)]. The likelihood of non-adherence was 3.57 times higher among adults who used the substance compared to those who were not users [AOR (95% CI) = 3.57 (1.86, 28.69)]. This study revealed that the odds of non-adherence among adults living at a distance greater than 10 km and above from ART clinic was more than two times higher compared to those residing near the ART-providing clinic [AOR (95% CI) = 2.42 (1.22, 25.86)]. Finally, patients whose medication time interfered with their daily routines were fifteen times more likely to be non-adherent compared to their counterparts [AOR (95% CI) = 15.46 (4.41, 54.28)] **(Table 4).**

**Table 4:** Factors associated with non-adherence among adult on first-line ART follow up at public health facilities of North Shewa Zone, Amhara Regional State, Ethiopia, 2019.

Variable Category		Adherence Status		COR (95%CI)	AOR (95% CI)
		Non-adhered	Adhered		
Educational level	No education	26 (48.2)	34 (13.3)	8.57 (4.06, 18.12)	5.57 (1.97, 15.68)*
	Primary	14 (25.9)	65 (25.4)	2.42 (1.09, 5.35)	2.19 (0.83, 5.76)
	Secondary and above	14 (25.9)	157 (61.3)	1.00	1.00
Monthly income	< 1500	24 (44.4)	76 (29.7)	1.00	1.00
	≥ 1500	30 (55.6)	180 (70.3)	052 (0.28, 0.96)	0.95 (0.37, 2.40)
Marital status	Married	14 (25.9)	76 (29.7)	1.00	1.00
	Unmarried	40 (74.1)	180 (70.3)	1.21 (0.62, 2.34)	0.52 (0.21, 1.26)
Residence	Urban	9 (16.7)	108 (42.2)	1.00	1.00
	Rural	45 (83.3)	148 (57.8)	3.65 (1.71, 7.78)	1.41 (0.13, 5.98)
Use memory aids to take ART drugs	Yes	14 (25.9)	155 (60.6)	1.00	1.00
	No	40 (74.1)	101 (39.4	4.38 (2.27, 8.46)	3.01 (1.27, 7.11)*
Get social support	Yes	11 (20.4)	110 (42.9)	1.00	1.00
	No	43 (79.6)	146 (57.1)	2.95(1.45, 5.97)	2.11 (0.84, 7.11)
Substance used in the last 7days	Yes	46 (85.2)	136 (53.1)	5.10 (2.30, 11.17)	3.57(1.86, 28.69)*
	No	8 (14.8)	120 (46.9)	1.00	1.00
Medication time interfere with daily activities	Yes	51 (16.5)	117 (37.7)	20.20(6.14,66.39)	15.46 (4.41,54.28)*
	No	3 (1.0)	139 (44.8)	1.00	1.00
Distance from ART clinic	<10killometer	10 (18.5)	109 (42.6)	1.00	1.00
	10killometrs and above	44 (81.5)	147 (57.6)	2.26 (1.57, 6.77)	2.42 (1.22, 25.86)*

* = statistically significant variables at 95%CI, COR = Crude Odds Ratio, AOR = Adjusted Odds Ratio, CI = Confidence Interval

## Discussion

Non-adherence to ART led to HIV-related morbidity and mortality [38, 39]. Our study revealed that ART non-adherence level based on self-report at the time of follow up was 17.4% (95% CI: 12.8%, 21.2%), higher than the findings of the studies in Southwest Ethiopia [17], northwest Ethiopia [20, 40], Eastern Ethiopia [22], Tanzania [41], South Africa [42], and Uganda [33]. The discrepancy could be due to differences in the study setting and the existence of socio-cultural differences of the participants.

However, the level of non-adherence in this study is lower than the findings of the studies in Southwest Ethiopia [19], Northeast Ethiopia [43], Southern Ethiopia [31], Papua New Guinea [44], and Nigeria [35]. The discrepancy might be explained by differences in the access to ART service, interventions taken [22], and awareness about the benefits of ART in the study areas.

In this study, the odds of non-adherence among adults who had no formal education was more than five times higher compared to those who attended secondary education and above. This finding is consistent with the result of the studies in Togo [45] and Guangzhou-China [24]. Higher education level has been one of the independent predictors to have adherence to ART [21, 22, 24, 42].

This study revealed that the odds of non-adherence among adults who used substances were more than three times higher compared to counterparts. This finding is supported by the studies conducted in Uganda [33], Vietnam [46], Togo [45], United States [47], Atlanta [48, 49], Amana Hospital, Tanzania [37], and Yaounde, Cameroon [50]. This will be can be explained by the fact that the use of substances like alcohol associated with memory impairment, subsequently failing patients to take the drugs as per schedule [34, 46, 49, 51–53]. It has also the potential to disrupt self-organizing skills and sleeping time patterns.

The odds of non-adherence among adults who did not use memory aids to take their ART drugs were three times higher compared to those who were using memory aids. This finding is in line with the study conducted in Jimma, Ethiopia [25], Vietnam [54], and Chicago [15]. The use of memory aid methods like peer counselors, mobile phone text messages, reminder devices, cognitive-behavioral therapy, and behavioral skills or medication adherence training [1, 5, 25, 55] have a great role in improving adherence. Thus, people using different reminders had a better level of adherence to the ART treatment [3, 56].

In this study, adults whose medication-taking time interfered with their daily routines had more than fifteen times higher odds of non-adherence compared to those whose medication schedules did not interfere with their daily activities. This finding is consistent with the findings of other studies [35, 56]. Hence, the people whose medication-taking time interfere with their working time are more likely to miss their ARV drugs which results in non-adherence [23, 31, 41].

In this study, the odds of non-adherence among adults living at a distance greater than 10 km and above from the ART clinic were more than two times higher compared to those residing nearer to the clinic. This finding is similar to the study conducted in Harari, Ethiopia [15], southwest Ethiopia [18], Indonesia [57], and the Democratic Republic [58]. This might be related to the availability of transportation and its costs. Thus, patients who live far away from the ART-providing clinics may not afford the transportation cost which leads to defaulting from the follow-up treatment.

As a limitation of the study, there might be recall bias since adherence was assessed based on data taken one month before the study period. Besides, the findings may not be generalized to patients who get ART services in private health facilities. However, to minimize recall bias we used triangulation of information from a medical record for some of the variables. In addition, due attention was given to all the study procedures throughout the field activities.

## Conclusions

In this study, the level of non-adherence to first-line ART was higher than the WHO standard. patients with no formal education, those who did not use memory aids to take their medications, travel more than 10 kilometers to visit the nearby ART clinics, those who used substance and patients whose medication time interfered with their daily routine activities had higher odds of having non-adherence to first-line ART compared to their counter groups.

Therefore, patients counseling focused on avoiding substance use, use memory aids, and adjusting working time with medication schedule are very crucial. Furthermore, the ministry of health and the regional health bureau with other stakeholders should expand ART service delivery at health facilities that are close to the community to address distance barriers.

## Supporting information

S1 Dataset Minimal dataset. (DTA)

## References

[1] USAIDS. Global HIV statistics, 2019. Available; https://www.unaids.org/en/resources/fact-sheet, accessed on march 12, 2020.

[2] USAIDS. Communities at the centre and global HIV/AIDS update 2019. Available; https://www.unaids.org/sites/default/files/media_asset/2019-global-AIDS-update_en.pdf, accessed on March 12, 2020.

[3] USAIDS. UNAIDS HIV/AIDS DATA, 2019. Available: https://www.unaids.org/en/resources/documents/2019/2019-UNAIDS-data, accessed on March 9, 2020.

[4] FMOH [Ethiopia]. HIV Prevention in Ethiopia National Road Map 2018–2020. Addis Ababa, Ethiopia, 2018.

[5] FMOH [Ethiopia]. National Consolidated Guidelines For Comprehensive Hiv Prevention, Care And Treatment. In. Addis Ababa, Ethiopia; 2018.

[6] Central Statistical Agency (CSA) [Ethiopia], ICF. Ethiopia Demographic and Health Survey 2016. Addis Ababa, Ethiopia, and Rockville, Maryland, USA: CSA and ICF. 2016.

[7] EPHI [Ethiopia]. HIV Related Estimates and Projections for 2017. In. Addis Ababa, Ethiopia; 2015.

[8] PBHIA [Ethiopia]. ETHIOPIA POPULATION-BASED HIV IMPACT ASSESSMENT 2017–2018. EPHI, CDC and PEPFAR. 2018.

[9] FMoFED [Ethiopia]. Assessing progress towards the Millenium Development Goals: Ethiopia MDGs report 2012. Addis Ababa, Ethiopia; 2012.

[10] FHAPCO [Ethiopia]. The country progress report on the HIV response. Addis Ababa; 2014.

[11] Mannheimer S, Friedland G, Matts J, Child C, Chesney M. Terry Beirn Community Programs for Clinical Research on AIDS. The consistency of adherence to antiretroviral therapy predicts biologic outcomes for Human Immunodeficiency Virus—infected persons in clinical trials. Clinical infectious diseases. 2002 Apr 15;34(8):1115–21. doi: 10.1086/339074 11915001

[12] Paterson DL, Swindells S, Mohr J, Brester M, Vergis EN, Squier C, et al. Adherence to protease inhibitor therapy and outcomes in patients with HIV infection. Annals of internal medicine. 2000 Jul 4;133(1):21–30. doi: 10.7326/0003-4819-133-1-200007040-00004 10877736

[13] Howard AA, Arnsten JH, Lo Y, Vlahov D, Rich JD, Schuman P, et al, HER Study Group. A prospective study of adherence and viral load in a large multi-center cohort of HIV-infected women. Aids. 2002 Nov 8;16(16):2175–82. doi: 10.1097/00002030-200211080-00010 12409739

[14] Nachega JB, Mills EJ, Schechter M. Antiretroviral therapy adherence and retention in care in middle-income and low-income countries: current status of knowledge and research priorities. Current Opinion in HIV and AIDS. 2010 Jan 1;5(1):70–7. doi: 10.1097/COH.0b013e328333ad61 20046150

[15] Mitiku H, Abdosh T, Teklemariam Z. Factors affecting adherence to antiretroviral treatment in harari national regional state, eastern ethiopia. *ISRN AIDS* 2013, 2013:960954. doi: 10.1155/2013/960954 24052892 PMC3773384

[16] Firdu N, Enquselassie F, Jerene D. HIV-infected adolescents have low adherence to antiretroviral therapy: a cross-sectional study in Addis Ababa, Ethiopia. *Pan Afr Med J* 2017, 27 (80). doi: 10.11604/pamj.2017.27.80.8544 28819501 PMC5554655

[17] Amberbir A, Woldemichael K, Getachew S, Girma B, Deribe K. Predictors of adherence to antiretroviral therapy among HIV-infected persons: a prospective study in Southwest Ethiopia. *BMC Public Health* 2008, 8:265. doi: 10.1186/1471-2458-8-265 18667066 PMC2518153

[18] Tiyou A, Belachew T, Alemseged F, Biadgilign S. Predictors of adherence to antiretroviral therapy among people living with HIV/AIDS in resource-limited setting of southwest ethiopia. *AIDS Res Ther* 2010, 7:39. doi: 10.1186/1742-6405-7-39 21034506 PMC2988692

[19] Molla AA, Gelagay AA, Mekonnen HS, Teshome DF. Adherence to antiretroviral therapy and associated factors among HIV positive adults attending care and treatment in University of Gondar Referral Hospital, Northwest Ethiopia. *BMC Infectious Diseases* 2018, 18(1):266. doi: 10.1186/s12879-018-3176-8 29879913 PMC5992657

[20] Dibaba B. aHM. Factors Associated with Non-Adherence to Antiretroviral Therapy among Adults living with HIV/AIDS in Arsi Zone, Oromia. *J AIDS Clin Res*: 2017, 8(1). 10.4172/2155-6113.1000652 PMC6103220 30140549

[21] O’Connor JL, Gardner EM, Mannheimer SB, Lifson AR, Esser S, Telzak EE, et al, Group ISS. Factors associated with adherence amongst 5295 people receiving antiretroviral therapy as part of an international trial. *J Infect Dis* 2013, 208(1):40–49. doi: 10.1093/infdis/jis731 23204161 PMC3666133

[22] Letta S, Demissie A, Oljira L, Dessie Y. Factors associated with adherence to Antiretroviral Therapy (ART) among adult people living with HIV and attending their clinical care, Eastern Ethiopia. *BMC Int Health Hum Rights* 2015, 15:33. doi: 10.1186/s12914-015-0071-x 26711659 PMC4693416

[23] Megerso A, Garoma S, Eticha T, Workineh T, Daba S, Tarekegn M, et al. Predictors of loss to follow-up in antiretroviral treatment for adult patients in the Oromia region, Ethiopia. *HIV AIDS (Auckl)* 2016, 8:83–92. doi: 10.2147/HIV.S98137 27175095 PMC4854271

[24] Muessig KE, McLaughlin MM, Nie JM, Cai W, Zheng H, Yang L, et al. Suboptimal antiretroviral therapy adherence among HIV-infected adults in Guangzhou, China. *AIDS Care* 2014, 26(8):988–995. doi: 10.1080/09540121.2014.897912 24666239 PMC4024070

[25] Bezabhe WM, Chalmers L, Bereznicki LR, Peterson GM, Bimirew MA, Kassie DM. Barriers and facilitators of adherence to antiretroviral drug therapy and retention in care among adult HIV-positive patients: a qualitative study from Ethiopia. *PLoS One* 2014, 9(5):e97353. doi: 10.1371/journal.pone.0097353 24828585 PMC4020856

[26] Kebede HK, Mwanri L, Ward P, Gesesew HA. Predictors of lost to follow up from antiretroviral therapy among adults in sub-Saharan Africa: a systematic review and meta-analysis. *Infectious diseases of poverty* 2021, 10(1):1–18 doi: 10.1186/s40249-020-00791-3 33743815 PMC7981932

[27] Federal Minstry of Health. Implementation of the Antiretroviral Therapy Programme in Ethiopia. 2007.

[28] Batuka JB, Yakobsen I, Meeting Targets and Maintaining Epidemic Control (EPIC) Project. Decentralized distribution of antiretroviral therapy through the private sector: a strategic guide for scale up. 2019.

[29] Reda AA, Biadgilign S. Determinants of Adherence to Antiretroviral Therapy among HIV-Infected Patients in Africa. *AIDS Res Treat* 2012, 2012:574656. doi: 10.1155/2012/574656 22461980 PMC3296173

[30] Somsit ANS, Benjaluck Phonrat T, Sirima Tepsupa LB. Early Viral Suppression Predicting Long-term Treatment Success Among HIV Patients Commencing NNRTI-based Antiretroviral Therapy. *Journal of Antivirals & Antiretrovirals* 2010, 02(02).

[31] Markos E, Worku A, Davey G. Adherence to ART in PLWHA and Yirgalem hospital, South Ethiopia. Ethiopian Journal of Health Development. 2008;22(2):174–9.

[32] UNAIDS. Global AIDS response progress reporting 2015. Geneva, Switherland; 2015.

[33] Shumba. C, Atuhaire. L, Imakit. R, Atukunda. R, Memiah. P: Missed Doses andMissed Appointments: Adherence to ART amongAdult Patients in Uganda. *Hindawi* 2013. 10.1155/2013/270914 PMC3767323 24052886

[34] Gebrezgabher BB, Kebede Y, Kindie M, Tetemke D, Abay M, Gelaw YA: Determinants to antiretroviral treatment non-adherence among adult HIV/AIDS patients in northern Ethiopia. *AIDS Res Ther* 2017, 14:16. doi: 10.1186/s12981-017-0143-1 28331527 PMC5359813

[35] Ijeoma Okoronkwo UO, Anthonia Chinweuba, and Peace Iheanacho. Nonadherence Factors and Sociodemographic Characteristics of HIV-Infected Adults Receiving Antiretroviral Therapy in Nnamdi Azikiwe University Teaching Hospital, Nnewi, Nigeria. Hindawi Publishing Corporation 2013. doi: 10.1155/2013/843794 PMC3863502 24369526

[36] Ivo N. Azia FCM, Brian van Wyk. Barriers to adherence to antiretroviral treatment in a regional hospital in Vredenburg, Western Cape, South Africa. *Southern African Journal of HIV Medicine* 2016, ISSN: (Online) 2078–6751, (Print) 1608–9693. 10.4102/sajhivmed.v17i1.476 PMC5843173 29568618

[37] Jima F, Tatiparthi R. Prevalence of nonadherence and its associated factors affecting on HIV adults follow-up at antiretroviral therapy clinic in Batu Hospital, Eastern Ethiopia. *Indian journal of sexually transmitted diseases and AIDS* 2018, 39(2):91. doi: 10.4103/ijstd.IJSTD_37_17 30623178 PMC6298155

[38] Legesse TA, Reta MA. Adherence to Antiretroviral Therapy and Associated Factors among People Living with HIV/AIDS in Hara Town and Its Surroundings, North-Eastern Ethiopia: A Cross-Sectional Study. *Ethiopian journal of health sciences* 2019, 29(3):299–308. doi: 10.4314/ejhs.v29i3.2 31447498 PMC6689727

[39] Lemma Negesa ED, and Wubshet Mekonnin. Adherence to Antiretroviral Therapy and Factors affecting among People Living with HIV/AIDS and Taking Antiretroviral Therapy, Dire Dawa Town, Eastern Ethiopia. *Journal of Infectious Diseases and Treatment*: 2017, 1(5).

[40] Tsega B, Srikanth BA, Shewamene Z. Determinants of non-adherence to antiretroviral therapy in adult hospitalized patients, Northwest Ethiopia. *Patient Prefer Adherence* 2015, 9:373–380. doi: 10.2147/PPA.S75876 25784793 PMC4356699

[41] Nyogea D, Mtenga S, Henning L, Franzeck FC, Glass TR, et al. Determinants of antiretroviral adherence among HIV positive children and teenagers in rural Tanzania: a mixed methods study. *BMC Infect Dis* 2015, 15:28. doi: 10.1186/s12879-015-0753-y 25637106 PMC4314748

[42] Mabunda K, Ngasama E, Babalola J, Zunza M, Nyasulu P. Determinants of adherence to antiretroviral treatment among human immunodeficiency virus infected young adults attending care at Letaba Hospital HIV Clinic, Limpopo Province, South Africa. *Pan African Medical Journal* 2019, 32(37). doi: 10.11604/pamj.2019.32.37.17722 31143342 PMC6522179

[43] Belayihun B, Negus R. Antiretroviral Treatment Adherence Rate and Associated Factors among People Living with HIV in Dubti Hospital, Afar Regional State, East Ethiopia. *Int Sch Res Notices* 2015, 2015:187360. doi: 10.1155/2015/187360 27347503 PMC4897121

[44] Gare J, Kelly-Hanku A, Ryan CE, David M, Kaima P, et al. Factors Influencing Antiretroviral Adherence and Virological Outcomes in People Living with HIV in the Highlands of Papua New Guinea. *PLoS One* 2015, 10(8):e0134918. doi: 10.1371/journal.pone.0134918 26244516 PMC4526685

[45] Issifou Yaya DEL, Bayaki Saka, P’Niwè Massoubayo Patchali, Peter Wasswa, et al. Predictors of adherence to antiretroviral therapy among people living with HIV and AIDS at the regional hospital of Sokodé, Togo. *BMC Public Health* 2014, 14(1308). 10.1186/1471-2458-14-1308 PMC4300825 25526773

[46] Bach Xuan Tran LTN, Cuong Duy Do, Quyen Le Nguyen and Rachel Marie Mahe. Associations between alcohol use disorders and adherence to antiretroviral treatment and quality of life amongst people living with HIV/AIDS. *BMC PUblic Health* 2014, 14(27). doi: 10.1186/1471-2458-14-27 24411007 PMC3893525

[47] Beer L, Skarbinski J. Adherence to antiretroviral therapy among HIV-infected adults in the United States. *AIDS Educ Prev* 2014, 26(6):521–537. doi: 10.1521/aeap.2014.26.6.521 25490733 PMC4579321

[48] Kalichman SC, Grebler T, Amaral CM, McKerney M, White D, et al. Food insecurity and antiretroviral adherence among HIV positive adults who drink alcohol. *J Behav Med* 2014, 37(5):1009–1018 doi: 10.1007/s10865-013-9536-3 24022091 PMC4283498

[49] Kalichman SC, Grebler T, Amaral CM, McNerey M, White D, Kalichman MO, et al. Intentional non-adherence to medications among HIV positive alcohol drinkers: prospective study of interactive toxicity beliefs. *J Gen Intern Med* 2013, 28(3):399–405. doi: 10.1007/s11606-012-2231-1 23065532 PMC3579979

[50] Pefura-Yone EW, Soh E, Kengne AP, Balkissou AD, Kuaban C. Non-adherence to antiretroviral therapy in Yaounde: prevalence, determinants and the concordance of two screening criteria. *J Infect Public Health* 2013, 6(4):307–315. doi: 10.1016/j.jiph.2013.02.003 23806707

[51] Chesney MA. Factors Affecting Adherence to Antiretroviral Therapy. *CID*: 2009, Supp 176. 10.1086/313849 10860902

[52] Pellowski JA, Kalichman SC, Kalichman MO, Cherry C. Alcohol-antiretroviral therapy interactive toxicity beliefs and daily medication adherence and alcohol use among people living with HIV. *AIDS Care* 2016, 28(8):963–970. doi: 10.1080/09540121.2016.1154134 26964014 PMC4963817

[53] Tessa Heestermans JLB, Susan C Aitken, Sigrid C Vervoort, Kerstin Klipstein-Grobusch. Determinants of adherence to antiretroviral therapy among HIV-positive adults in sub-Saharan Africa: a systematic review. *BMJ Glob Health*: 2016, 1(e000125.). doi: 10.1136/bmjgh-2016-000125 28588979 PMC5321378

[54] Tran BX, Nguyen LT, Nguyen NH, Hoang QV, Hwang J. Determinants of antiretroviral treatment adherence among HIV/AIDS patients: a multisite study. *Glob Health Action* 2013, 6:19570. doi: 10.3402/gha.v6i0.19570 23497956 PMC3600425

[55] USAIDS. GLOBAL HIV/AIDS UPDATE 2019. Available; https://www.unaids.org/sites/default/files/media_asset/2019-global-AIDS-update_en.pdf, accessed on March 12, 2020.

[56] Axelsson JM, Hallager S, Barfod TS. Antiretroviral therapy adherence strategies used by patients of a large HIV clinic in Lesotho. *J Health Popul Nutr* 2015, 33:10. doi: 10.1186/s41043-015-0026-9 26825572 PMC5025960

[57] Weaver ER, Pane M, Wandra T, Windiyaningsih C, Herlina, Samaan G. Factors that influence adherence to antiretroviral treatment in an urban population, Jakarta, Indonesia. *PLoS One* 2014, 9(9):e107543. 25229671 10.1371/journal.pone.0107543 PMC4168004

[58] Visanou Hansana PS, Jo Durham, Vanphanom Sychareun, Kongmany Chaleunvong, Suwanna Boonyaleepun and Frank Peter Schelp. Adherence to Antiretroviral Therapy (ART) among People Living With HIV (PLHIV): a cross-sectional survey to measure in Lao PDR. *BMC Public Health* 2013, 13(617). doi: 10.1186/1471-2458-13-617 23809431 PMC3707741

